# Interactions between genetic variants involved in the folate metabolic pathway and serum lipid, homocysteine levels on the risk of recurrent spontaneous abortion

**DOI:** 10.1186/s12944-019-1083-7

**Published:** 2019-06-15

**Authors:** Zhong Lin, Qianxi Li, Yifan Sun, Jingchun Huang, Wan Wang, Jinjian Fu, Jianhua Xu, Dingyuan Zeng

**Affiliations:** 1 Department of Obstetrics and Gynecology, Liuzhou Maternity and Child Health Care Hospital, 50 Yingshan Road, Liuzhou, 545001 Guangxi China; 2 Department of Obstetrics and Gynecology, The Maternal & Child Health Hospital of Guangxi Zhuang Autonomous Region, Guangxi, 530003 China; 3 Department of Clinical Laboratory, Affiliated Liutie Central Hospital of Guangxi Medical University, Liuzhou, 545001 Guangxi China; 4 Department of Laboratory Science, The Second Affiliated Hospital of Guangzhou University of Chinese Medicine, Guangzhou, 510120 Guangdong China; 5 Department of Laboratory, Liuzhou Maternity and Child Health Care Hospital, Liuzhou, 545001 Guangxi China; 6 Laboratory of Oncology Science and Molecular Biology, ShunDe Hospital of Guangzhou University of Chinese Medicine, Shunde, 528333 Guangdong China

**Keywords:** MTHFR C677T, MTHFR A1298C, MTRR A66G, Homocysteine, Lipid profiles, Recurrent spontaneous abortion

## Abstract

**Background:**

The interaction between folate pathway gene polymorphisms and homocysteine, serum lipid leverls are poorly understood in patients with recurrent spontaneous abortion (RSA). The aim of this study is to explore the effects of folate pathway gene polymorphisms (the 5–10-methylenetetrahydrofolate reductase, MTHTR C677T, MTHFR A1298C and the methionine synthase reductase, MTRR A66G) and their interactions with homocysteine on serum lipid levels in patients with RSA.

**Methods:**

A total of 403 RSA women and 342 healthy women were randomly selected. Genotyping of the MTHFR C677T, A1298C and MTRR A66G were performed by TaqMan-MGB technique. Serum homocysteine, folate, fasting glucose, fasting insulin, Interleukin 6, Tumor necrosis factorα (TNFα) and lipid profiles were measured according to the kits. Continuous variables were analyzed using 2-sample t-tests. Categorical variables were analyzed and compared by *χ*^2^ or Fisher’s exact tests. Unconditional logistic regression model was applied to test the interactions of gene polymorphisms on RSA.

**Results:**

The distribution of genotype of CC, CT TT and T allele of MTHFR C677T, genotype of AA and C allele of MTHFR A1298C, and genotype of AA, AG and G allele of MTRR A66G were different between cases and controls (all *p* were < 0.05). There were significant interactions between MTHFR C677T-A1298C and MTHFR A1298C-MTRR A66G in RSA group and control group, with ORs of 1.62 (95%CI: 1.28–2.04, *p* < 0.001) and 1.55 (95%CI: 1.27–1.88, *p* < 0.001), respectively. Serum TNFα level and insulin resistant status (HOMR-IR) were higher in RSA group than in control group (*p* = 0.038, 0.001, respectively). All the three gene SNPs except MTRR 66AG gene variant had detrimental effects on HOMA-IR (all *p* were < 0.05). RSA group who carried the MTHFR 677CT, TT, CT/TT genotypes and MTRR 66AG, AG/GG genotypes had detrimental effects on serum homocysteine levels, the MTHFR 677CT, CT/TT genotype carriers had favorable effects on serum folate levels, the MTHFR 677TT, CT/TT, 1298 AC, AC/CC genotype carriers had detrimental effects on serum low-density lipoprotein cholesterol (LDL-C) levels, and the MTRR 66AG genotype carriers had lower high-density lipoprotein cholesterol (HDL-C) levels than the AA genotype carriers (all *p* were < 0.05).

**Conclusions:**

Interaction between the MTHFR C677T, A1298C and MTHFR A1298C, MTRR A66G are observed in our RSA group. Besides, all the three gene SNPs except MTRR 66AG gene variant had detrimental effects on HOMA-IR. MTHFR C677T and MTRR A66G gene variants had detrimental effects on serum homocysteine levels and insulin resistance status, while MTHFR C677T, A1298C and MTRR A66G gene variants had detrimental effects on certain serum lipid profiles.

## Background

Recurrent spontaneous abortion (RSA) is a common health problem, defined as the loss of two or more consecutive pregnancies before 20 weeks of gestation which is challenging for both the patients and obstetricians [1]. RSA is a complex multi-factorial disorder and caused very often by genetic disorders, uterine pathologies, endocrine dysfunctions, autoimmune diseases, and environmental factors [1]. Dyslipidemia has been postulated as association with adverse pregnancy outcome, including RSA [2].

Dyslipidemia, as mainly defined by increased serum total cholesterol (TC) and low density lipoprotein cholesterol (LDL-C) levels, serving as a crucial risk factor for some medical diseases such as cardiovascular diseases, diabetes and insulin resistance, has become a serious public health problem worldwide because of its high prevalence [3–5]. It was reported that the prevalence of dyslipidemia among Chinese adults increases yearly and the prevalence of dyslipidemia was 52.72% among adults in northwestern China in 2010 [6]. The etiology of dyslipidemia is complicated, both genetic and environmental factors as well as their interactions are considered to be the contributors for the cause of dyslipidemia [7, 8].

The 5–10-methylenetetrahydrofolate reductase (MTHFR) C677T and A1298C and methionine synthase reductase (MTRR) A66G gene, may contribute to the risk of the development of hyperhomocysteinemia and are now believed to be good candidate for susceptibility to dyslipidemia and insulin resistance [9, 10]. Numerous epidemiological studies revealed that high homocysteine levels have been suggested to be associated with changing serum lipid levels.

Recent attention has focused on certain gene polymorphism and biomarkers interaction that may predispose to an increased risk of severe pregnancy complications, including RSA [11]. Only recently genetic analyses of affected patients was it discovered that C677T, A1298C polymorphisms of MTHFR and A66G of MTRR may represent the important candidates for exploration of the risk of developing disease as their key roles for not only in gene expression but also in modifications of serum lipid and homocystein concentrations [12].

Few studies so far have investigated the effect of homocysteine, insulin resistance, TNFαand lipid levels and the MTHFR, MTRR gene polymorphisms on RSA risk. Mtiraoui et al. [13] have demonstrated that MTHFR gene polymorphisms were associated with progression of recurrent miscarriage through elevations of plasma homocysteine levels. Ikkruthi et al. [14] have revealed that hyperhomocysteinemia was associated with hyperlipoproleinemia. Li et al. [9] identified that MTHFR C677T, A1298C and MTRR A66G gene polymorphisms combined with low folate were the major determinant of plasma lipid levels.

In summary, elevated plasma levels of homocysteine may cause RSA and dysregulation of cholesterol and triglyceride biosynthetic pathways, with changed expression by DNA methylation. As a consequence, we hypothesize that the MTHFR and MTRR gene polymorphisms associated with higher levels of homocysteine may be related with different serum lipid levels in the RSA populations. The aim of this study is to explore the interactions of these three gene polymorphisms (MTHFR C677T, MTHFR A1298C and MTRR A66G), homocysteine and serum lipid profiles with RSA in Chinese population.

## Methods

### Study population

This investigation was carried out as a case–control study conducted between January 1, 2013 and November 12, 2015, in the Gynecology clinic of Liuzhou Maternity and Child Healthcare Hospital. A total of 403 women who had 2 or more consecutive spontaneous abortions were diagnosed as RSA and recruited as case group. Control group consisted of 342 healthy women of reproductive age with at least 1 delivery and no history of abortion. Women who had chromosomal abnormalities, personal or family history of thrombosis, induced abortions, infection or systemic diseases were excluded from this study. A questionnaire detailing age, ethnic, education level, gynecological history, smoking, drinking, X-ray contact, chemical exposure, folate supplement, multivitamin supplement were asked to fill and consent form indicating their acceptance to participate were signed and obtained. This study was approved by the Institutional Review Board at Liuzhou Maternity and Child Healthcare Hospital.

### Laboratory tests

EDTA-anticoagulated blood (5 ml sample) and buccal cell samples were obtained from participants and was processed within 30 min of collection for biochemical analysis and genetic analysis, respectively. The levels of triglyceride (TG), TC, high-density lipoprotein cholesterol (HDL-C), LDL-C, total protein, homocysteine and fast glucose in blood samples were measured by enzymatic method on a Hitachi Autoanalyzer (Type 7600; Hitachi Ltd., Tokyo, Japan). The levels of folate, vitamin B12 and fast insulin in blood samples were measured by chemi-luminescence method on a Abbott Autoanalyzer (Type i4000SR; Abbott Ltd., America). The levels of IL6 and TNFα were measured by liquid suspension chip on luminex200 (Austin, Texas, America).

### Genotyping

Genomic DNA was extracted from buccal samples using the QIAamp DNA Mini Kit (Qiagen, Valencia, CA, USA). The TaqMan-MGB technique was used for detecting gene polymorphisms of the MTHFR C677T, A1298C and MTRR A66G. The primers and probes were showed in Table 1. Universal reaction conditions in a final volume of 10 μl for each genotyping are as follows: 1 μl of 20 ng/μl DNA, 5 μl of 2 × Taqman Universal Master Mix, 0.5 μl of 20 × TaqMan-MGB assay locus-specific probe, with 3.5 μl of sterile water. All PCR reagents were purchased from ABI Company. The PCR cycling conditions were 1 cycle of 95 °C for 10 min; then 20 cycles of 96 °C for 15 s, 60 °C for 60 s; then 30 cycles of 89 °C for 15 s, 60 °C for 60 s. After PCR amplification, an endpoint plate read was performed using an Applied Biosystems Real-Time PCR System. The Sequence Detection System (SDS) Software uses the fluorescence measurements made during the plate read to plot the fluorescence (Rn) values based on the signals from each well. The plotted fluorescence signals indicate the alleles that are present in each sample. All cycling protocols were performed on a ABI 7900.

**Table 1:** TaqMan-MGB primers and probes

SNPs	Primers		Probes	
	Forward	Reverse	Forward	Reverse
MTHFR C677T	GAAAAGCTGCGTGATGATG	TTGAAGGAGAAGGTGTC	AATCG [G]CTCCCGC	AATCG [A]CTCCCGC
MTHFR A1298C	AAGAACGAAGACTTCAAA	TGGGGGGAGGAGCTGAC	ACACTT [G]CTTCACT	ACACTT [T]CTTCACT
MTRR A66G	AGGCAAAGGCCATCGCA	ATCCATGTACCACAGCTT	AAGAAAT [A]TGTGAG	AAGAAAT [G]TGTGAG

### Statistical analysis

SAS version 9.4 (Cary, NC, USA) was used to perform the statistical analysis. Continuous variables were analyzed using 2-sample *t*-tests. Categorical variables were analyzed and compared by *χ*^2^ or Fisher’s exact tests. For the main effect of gene-gene variants interactions, unconditional logistic regression was conducted to calculate odds ratios (ORs) and their corresponding 95% confidence intervals (CIs). A *p*-value less than 0.05 was considered indicative of statistical significance.

## Results

### General characteristics, serum lipid levels and allelic frequencies

Table 2 examined the characteristics, homocysteine, serum lipid levels and allelic frequencies of MTHFR C677T, A1298C and MTRR A66G between the RSA group and healthy group. A *χ*^2^ analysis has found that folate supplement was higher in control group than in case group (*p* = 0.048). HOMA-IR index wes higher in the RSA group than in the control group (*p* = 0.001). The levels of homocysteine, serum total protein, LDL-C and TNFα were higher in RSA group than in control group (all *p* were < 0.05), whereas the level of HDL-C was lower in RSA group than in control group (*p* = 0.018).

**Table 2:** General characteristics and genotype distribution

Variable	Case (*n* = 403)	Control (*n* = 342)	*t/χ* ^2^	*p*
Ethnic			0.43	0.805
Han	198 (49.1)	160 (46.8)		
Zhuang	178 (44.2)	159 (46.5)		
Minority	27 (6.7)	23 (6.7)		
Education level			1.20	0.550
≤ 9 years of school	157 (39.0)	120 (35.1)		
10–12 years of school	83 (20.6)	74 (21.6)		
≥ 13 years of school	163 (40.4)	148 (43.3)		
Gynecological surgery history			1.09	0.298
Yes	42 (10.4)	28 (8.2)		
No	361 (89.6)	314 (91.8)		
Current smoking			0.10	0.756
Yes	7 (1.7)	7 (2.0)		
No	396 (98.3)	335 (98.0)		
Passive smoking history			0.003	0.960
Yes	89 (22.1)	75 (21.9)		
No	314 (77.9)	267 (78.1)		
Drinking history			0.04	0.847
Yes	43 (10.7)	38 (11.1)		
No	360 (89.3)	304 (88.9)		
X-ray contact history			0.52	0.596
Yes	1 (0.2)	2 (0.6)		
No	402 (99.8)	340 (99.4)		
Chemical exposure history			3.41	0.065
Yes	1 (0.2)	5 (1.5)		
No	402 (99.8)	337 (98.5)		
Folic acid supplement			3.91	0.048
Yes	131 (32.5)	135 (39.5)		
No	272 (67.5)	207 (60.5)		
Multivitamin supplement			1.38	0.240
Yes	17 (4.2)	9 (2.6)		
No	386 (95.8)	333 (97.4)		
Age, year	29.58 ± 5.48	29.88 ± 5.28	0.85	0.673
Folic acid, nmol/L	32.45 ± 8.86	34.35 ± 18.98	−1.79	0.072
Vitamin B12, pg/ml	352.70 ± 124.01	347.48 ± 124.10	0.57	0.567
Homocysteine, umol/L	11.89 ± 4.62	11.20 ± 3.40	2.36	0.018
Total protein, g/L	72.17 ± 6.79	70.67 ± 9.47	2.52	0.012
Total cholesterol, mmol/L	5.43 ± 20.10	4.42 ± 1.91	0.92	0.359
Triglyceride, mmol/L	1.06 ± 0.69	1.03 ± 0.55	0.62	0.536
High-density lipoprotein cholesterol, mmol/L	1.66 ± 0.38	1.73 ± 0.38	−2.38	0.018
Low-density lipoprotein cholesterol, mmol/L	2.61 ± 0.77	2.41 ± 0.68	3.62	< 0.001
Fasting glucose, mmol/L	4.93 ± 0.38	4.92 ± 0.19	0.15	0.884
Fasting insulin, pmol/L	71.13 ± 14.82	67.60 ± 13.84	3.35	0.001
HOMR-IR	2.23 ± 0.45	2.12 ± 0.41	3.48	0.001
IL6, pg/ml	75.12 ± 311.20	40.58 ± 202.95	1.82	0.069
TNFα, pg/ml	28.40 ± 92.16	18.28 ± 31.02	2.08	0.038
MTHFR C677T				
CC	213 (52.9)	253 (74.0)	35.24	< 0.001
CT	153 (38.0)	78 (22.8)	19.87	< 0.001
TT	37 (9.2)	11 (3.2)	10.92	0.001
C allele	579 (71.8)	584 (85.4)	–	–
T allele	227 (28.2)	100 (14.6)	39.62	< 0.001
MTHFR A1298C				
AA	231 (57.3)	221 (64.6)	4.13	0.042
AC	144 (35.7)	102 (29.8)	2.92	0.088
CC	28 (6.9)	19 (5.6)	0.61	0.436
A allele	606 (75.2)	544 (79.5)	–	–
C allele	200 (24.8)	140 (20.5)	3.97	0.046
MTRR A66G				
AA	225 (55.8)	226 (66.1)	8.14	0.004
AG	148 (36.7)	100 (29.2)	4.67	0.031
GG	30 (7.4)	16 (4.7)	2.44	0.118
A allele	598 (74.2)	552 (80.7)	–	–
G allele	208 (25.8)	132 (19.3)	8.90	0.003

*HOMA-IR* Homeostatic model assessment of insulin resistance, *IL6* Interleukin 6, *TNFα* Tumor necrosis factor α

The frequency of MTHFR C677T, A1298C and MTRR A66G alleles and genotypes are shown in Table 2. The frequencies of CC, CT and TT genotypes and T allele of C677T were 0.529, 0.380, 0.092 and 0.282 in cases, compared with 0.740, 0.228, 0.032 and 0.146 in controls, respectively (*p* < 0.001–0.001). The distribution of genotype of AA and C allele of MTHFR A1298C were slightly different between cases and controls (*p* = 0.042 and 0.046, respectively). The distribution of genotype of AA, AG and G allele of MTRR A66G were different between cases and controls (*p* = 0.004, 0.0031 and 0.003, respectively).

The two-factor gene-gene interaction analyses by logistic regression analysis revealed significant interactions between MTHFR C677T-A1298C and MTHFR A1298C-MTRR A66G in RSA group and control group, with ORs of 1.62 (95%CI: 1.28–2.04, *p* < 0.001) and 1.55 (95%CI, 1.27–1.88, *p* < 0.001), respectively (Table 3).

**Table 3:** Interactions between genetic variants in the folate pathway on the risk of recurrent spontaneous abortion

Gen-gen interactions	B	SE	Wald	*p*	OR	95%CI
C677T-A1298C	0.48	0.12	16.23	0.000	1.62	1.28–2.04
C677T-A66G	0.02	0.08	0.07	0.799	1.02	0.87–1.20
A1298C-A66G	0.44	0.10	18.84	0.000	1.55	1.27–1.88

### MTHFR C677T genotypes and serum homocysteine, inflammatory factor and lipid levels

Table 4 shows the interaction of MTHFR C677T gene polymorphism with RSA risk on serum homocysteine, inflammatory factor and lipid levels. All the three gene variants had detrimental effects on HOMA-IR (all *p* were < 0.05). The CT genotype carriers had higher serum homocysteine levels and lower folate levels in the RSA group than that in the control group (*p* < 0.001 and 0.047, respectively). The RSA group who carrying TT genotype had higher serum homocysteine and LDL-C levels than that in the control group (*p* = 0.026 and 0.006, respectively). For those RSA group who carried CT/TT genotype, they had higher serum homocysteine and LDL-C levels and lower folate levels than that in the control group (*p* = 0.003, 0.018 and 0.012, respectively).

**Table 4:** Interaction of MTHFR C677T polymorphism with recurrent spontaneous abortion on serum folate and lipid levels

Variable	Case	control	*t*	*p*
CC genotype	*n* = 213	*n* = 253		
Folic acid, nmol/L	36.49 ± 6.47	35.29 ± 21.53	0.78	0.434
Vitamin B12, pg/ml	347.23 ± 114.82	346.84 ± 124.51	0.03	0.976
Homocysteine, umol/L	10.33 ± 4.34	10.83 ± 3.38	−1.39	0.165
Total protein, g/L	72.87 ± 4.78	70.69 ± 9.99	2.93	0.004
Total cholesterol, mmol/L	4.48 ± 1.09	4.51 ± 2.13	−0.16	0.873
Triglyceride, mmol/L	1.06 ± 0.81	1.03 ± 0.57	0.68	0.494
High-density lipoprotein cholesterol, mmol/L	1.68 ± 0.42	1.74 ± 0.36	−1.81	0.071
Low-density lipoprotein cholesterol, mmol/L	2.65 ± 0.84	2.16 ± 0.60	1.78	0.082
Fasting glucose, mmol/L	4.93 ± 0.38	4.92 ± 0.29	0.26	0.798
Fasting insulin, pmol/L	70.64 ± 14.43	67.90 ± 14.03	2.08	0.038
HOMR-IR	2.21 ± 0.45	2.12 ± 0.42	2.12	0.035
IL6, pg/ml	81.84 ± 339.77	33.09 ± 215.37	1.89	0.058
TNFα, pg/ml	29.11 ± 88.72	18.86 ± 30.27	1.75	0.081
CT genotype	*n* = 153	*n* = 78		
Folic acid, nmol/L	32.73 ± 7.85	38.43 ± 9.29	−3.55	0.000
Vitamin B12, pg/ml	359.98 ± 131.88	353.94 ± 115.88	0.33	0.745
Homocysteine, umol/L	12.65 ± 3.97	11.71 ± 3.06	1.99	0.047
Total protein, g/L	71.68 ± 8.05	70.61 ± 8.01	0.92	0.361
Total cholesterol, mmol/L	6.99 ± 32.56	4.17 ± 0.81	0.72	0.475
Triglyceride, mmol/L	1.02 ± 0.52	0.95 ± 0.37	0.97	0.332
High-density lipoprotein cholesterol, mmol/L	1.67 ± 0.33	1.72 ± 0.48	−0.92	0.358
Low-density lipoprotein cholesterol, mmol/L	2.59 ± 0.78	2.39 ± 0.74	1.82	0.070
Fasting glucose, mmol/L	4.95 ± 0.37	4.88 ± 0.39	0.62	0.532
Fasting insulin, pmol/L	72.28 ± 15.42	66.89 ± 12.57	2.86	0.005
HOMR-IR	2.27 ± 0.47	2.11 ± 0.36	2.83	0.005
IL6, pg/ml	72.60 ± 275.52	41.08 ± 147.43	1.09	0.276
TNFα, pg/ml	29.34 ± 106.30	16.52 ± 34.81	1.25	0.210
TT genotype	*n* = 37	*n* = 11		
Folic acid, nmol/L	25.72 ± 7.67	24.54 ± 7.84	0.45	0.658
Vitamin B12, pg/ml	354.14 ± 142.24	322.82 ± 167.57	0.62	0.541
Homocysteine, umol/L	17.80 ± 2.86	15.93 ± 2.08	2.39	0.026
Total protein, g/L	70.17 ± 9.96	70.42 ± 3.79	−0.08	0.933
Total cholesterol, mmol/L	4.34 ± 1.01	3.96 ± 0.80	1.13	0.265
Triglyceride, mmol/L	1.11 ± 0.57	1.49 ± 0.73	−1.85	0.071
High-density lipoprotein cholesterol, mmol/L	1.58 ± 0.33	1.62 ± 0.26	−0.31	0.761
Low-density lipoprotein cholesterol, mmol/L	2.61 ± 0.76	2.43 ± 0.67	2.78	0.006
Fasting glucose, mmol/L	4.95 ± 0.43	4.79 ± 0.36	1.38	0.175
Fasting insulin, pmol/L	70.52 ± 16.13	67.86 ± 17.26	0.55	0.583
HOMR-IR	2.21 ± 0.45	2.07 ± 0.54	0.96	0.341
IL6, pg/ml	14.94 ± 43.01	104.44 ± 273.61	−1.58	0.126
TNFα, pg/ml	15.72 ± 29.57	20.42 ± 19.78	−0.65	0.521
CT /TTgenotype	*n* = 190	*n* = 89		
Folic acid, nmol/L	31.31 ± 8.99	35.11 ± 21.36	−2.96	0.003
Vitamin B12, pg/ml	358.84 ± 133.59	349.61 ± 123.50	0.53	0.598
Homocysteine, umol/L	12.37 ± 4.57	11.52 ± 3.65	2.52	0.012
Total protein, g/L	71.39 ± 8.45	70.59 ± 7.55	0.73	0.465
Total cholesterol, mmol/L	6.48 ± 29.22	4.14 ± 0.81	0.71	0.478
Triglyceride, mmol/L	1.04 ± 0.53	1.03 ± 0.47	0.14	0.887
High-density lipoprotein cholesterol, mmol/L	1.66 ± 0.34	1.71 ± 0.45	−1.08	0.281
Low-density lipoprotein cholesterol, mmol/L	2.61 ± 0.79	2.36 ± 0.72	2.38	0.018
Fasting glucose, mmol/L	4.95 ± 0.38	4.94 ± 0.39	0.15	0.158
Fasting insulin, pmol/L	71.99 ± 15.50	67.08 ± 13.55	2.78	0.006
HOMR-IR	2.26 ± 0.47	2.11 ± 0.40	2.96	0.003
IL6, pg/ml	63.25 ± 253.50	53.54 ± 119.70	0.37	0.714
TNFα, pg/ml	15.72 ± 29.57	20.42 ± 19.78	−0.65	0.521

*HOMA-IR* Homeostatic model assessment of insulin resistance, *IL6* Interleukin 6, *TNFα*: Tumor necrosis factor α

### MTHFR A1298C genotypes and serum homocysteine, lipid levels

Table 5 shows the interaction of MTHFR A1298C gene polymorphism with RSA risk on serum homocysteine and lipid levels. All the three gene variants had detrimental effects on HOMA-IR (all *p* were < 0.05). The AA genotype carriers had lower HDL-C levels in the RSA group than that in the control group (*p* = 0.012). The RSA group who carrying AC genotype had higher serum LDL-C levels than that in the control group (*p* < 0.001). For RSA cases who carried AC/CC genotype, they had higher serum LDL-C levels than that in the control group (*p* < 0.001).

**Table 5:** Interaction of MTHFR A1298C polymorphism with recurrent spontaneous abortion on serum folate and lipid levels

Variable	Case	control	*t*	*p*
AA genotype	*n* = 231	*n* = 221		
Folic acid, nmol/L	31.28 ± 9.77	33.89 ± 23.08	−1.58	0.115
Vitamin B12, pg/ml	352.21 ± 125.06	347.58 ± 124.71	0.39	0.694
Homocysteine, umol/L	11.93 ± 4.63	11.27 ± 2.75	1.81	0.071
Total protein, g/L	71.91 ± 7.99	70.26 ± 10.93	1.83	0.067
Total cholesterol, mmol/L	4.32 ± 0.84	4.47 ± 2.08	−1.05	0.295
Triglyceride, mmol/L	1.07 ± 0.77	1.06 ± 0.59	0.13	0.894
High-density lipoprotein cholesterol, mmol/L	1.67 ± 0.39	1.76 ± 0.40	−2.51	0.012
Low-density lipoprotein cholesterol, mmol/L	2.56 ± 0.74	2.45 ± 0.71	1.65	0.101
Fasting glucose, mmol/L	4.92 ± 0.37	4.92 ± 0.38	0.02	0.987
Fasting insulin, pmol/L	72.88 ± 14.93	67.83 ± 14.42	3.55	< 0.001
HOMR-IR	2.28 ± 0.46	2.12 ± 0.43	3.74	< 0.001
IL6, pg/ml	58.24 ± 239.93	36.23 ± 138.03	1.19	0.235
TNFα, pg/ml	29.47 ± 108.57	19.73 ± 35.54	1.29	0.198
AC genotype	*n* = 144	*n* = 102		
Folic acid, nmol/L	34.18 ± 6.80	35.24 ± 6.50	−1.23	0.218
Vitamin B12, pg/ml	354.39 ± 129.77	350.37 ± 116.89	0.25	0.804
Homocysteine, umol/L	11.85 ± 4.63	11.06 ± 4.35	1.49	0.137
Total protein, g/L	72.69 ± 4.52	71.63 ± 6.12	1.52	0.129
Total cholesterol, mmol/L	7.41 ± 33.55	4.21 ± 1.23	0.96	0.337
Triglyceride, mmol/L	1.07 ± 0.59	0.96 ± 0.44	1.54	0.126
High-density lipoprotein cholesterol, mmol/L	1.65 ± 0.36	1.70 ± 0.35	−1.15	0.252
Low-density lipoprotein cholesterol, mmol/L	2.73 ± 0.83	2.34 ± 0.65	3.88	0.000
Fasting glucose, mmol/L	4.95 ± 0.39	4.94 ± 0.42	0.16	0.873
Fasting insulin, pmol/L	68.94 ± 14.64	67.46 ± 13.49	0.03	0.409
HOMR-IR	2.16 ± 0.43	2.11 ± 0.40	0.89	0.372
IL6, pg/ml	109.82 ± 422.09	51.87 ± 305.40	1.26	0.208
TNFα, pg/ml	28.91 ± 69.92	15.59 ± 21.63	2.16	0.032
CC genotype	*n* = 28	*n* = 19		
Folic acid, nmol/L	33.18 ± 9.09	34.99 ± 8.43	−0.69	0.494
Vitamin B12, pg/ml	348.11 ± 80.49	330.79 ± 157.10	0.50	0.622
Homocysteine, umol/L	12.43 ± 5.99	11.84 ± 4.58	0.36	0.717
Total protein, g/L	71.67 ± 4.22	70.19 ± 4.62	1.14	0.262
Total cholesterol, mmol/L	4.27 ± 0.74	4.93 ± 2.66	−1.23	0.226
Triglyceride, mmol/L	0.89 ± 0.37	1.00 ± 0.49	−0.91	0.366
High-density lipoprotein cholesterol, mmol/L	1.78 ± 0.42	1.62 ± 0.32	1.44	0.156
Low-density lipoprotein cholesterol, mmol/L	2.41 ± 0.67	2.38 ± 0.53	0.16	0.876
Fasting glucose, mmol/L	4.93 ± 0.38	5.00 ± 0.35	−0.81	0.420
Fasting insulin, pmol/L	68.09 ± 13.33	66.68 ± 11.63	0.45	0.657
HOMR-IR	2.14 ± 0.46	2.12 ± 0.36	0.24	0.809
IL6, pg/ml	37.96 ± 81.36	30.61 ± 91.73	0.34	0.735
TNFα, pg/ml	17.62 ± 22.33	18.33 ± 27.97	−0.11	0.910
AC /CCgenotype	*n* = 172	*n* = 121		
Folic acid, nmol/L	34.02 ± 7.20	35.20 ± 6.80	−1.42	0.155
Vitamin B12, pg/ml	353.37 ± 122.93	347.30 ± 23.50	0.42	0.678
Homocysteine, umol/L	11.86 ± 4.63	11.06 ± 4.35	1.49	0.137
Total protein, g/L	72.52 ± 4.73	71.40 ± 5.92	1.80	0.073
Total cholesterol, mmol/L	6.92 ± 30.79	4.32 ± 1.55	0.93	0.356
Triglyceride, mmol/L	1.04 ± 0.57	0.96 ± 0.45	1.14	0.253
High-density lipoprotein cholesterol, mmol/L	1.67 ± 0.37	1.68 ± 0.35	−0.42	0.672
Low-density lipoprotein cholesterol, mmol/L	2.67 ± 0.81	2.35 ± 0.63	3.69	0.000
Fasting glucose, mmol/L	4.94 ± 0.39	4.95 ± 0.40	−0.22	0.826
Fasting insulin, pmol/L	68.79 ± 14.39	67.27 ± 13.02	0.98	0.325
HOMR-IR	2.16 ± 0.43	2.11 ± 0.39	0.93	0.351
IL6, pg/ml	97.57 ± 386.58	46.67 ± 269.04	1.37	0.170
TNFα, pg/ml	26.98 ± 64.43	16.26 ± 23.26	2.04	0.042

*HOMA-IR* Homeostatic model assessment of insulin resistance, *IL6* Interleukin 6, *TNFα* Tumor necrosis factor α

### MTRR A66G genotypes and serum homocysteine, lipid levels

Table 6 shows the interaction of MTRR A66G gene polymorphism with RSA risk on serum homocysteine and lipid levels. The AA genotype carriers had higher HOMA-IR, total protein and LDL-C levels in the RSA group than that in the control group (*p* = 0.011, 0.008 and < 0.001, respectively). The RSA group who carrying AG genotype had higher serum homocysteine levels and lower serum HDL-C levels than that in the control group (*p* = 0.047 and 0.010, respectively). For RSA patients who carried AG/GG genotype, they had higher HOMA-IR, serum homocysteine levels than that in the control group (*p* = 0.020 and 0.030, respectively).

**Table 6:** Interaction of MTRR A66G polymorphism with recurrent spontaneous abortion on serum folate and lipid levels

Variable	Case	control	*t*	*p*
AA genotype	*n* = 225	*n* = 226		
Folic acid, nmol/L	33.20 ± 9.06	33.58 ± 8.42	−0.57	0.572
Vitamin B12, pg/ml	362.49 ± 134.76	346.12 ± 126.65	1.33	0.185
Homocysteine, umol/L	11.38 ± 4.59	11.05 ± 3.49	0.86	0.393
Total protein, g/L	72.83 ± 5.21	71.08 ± 8.41	2.64	0.008
Total cholesterol, mmol/L	6.27 ± 26.85	4.48 ± 2.25	0.99	0.320
Triglyceride, mmol/L	1.17 ± 0.55	1.05 ± 0.56	1.75	0.081
High-density lipoprotein cholesterol, mmol/L	1.68 ± 0.41	1.72 ± 0.39	−1.15	0.251
Low-density lipoprotein cholesterol, mmol/L	2.67 ± 0.79	2.39 ± 0.72	3.97	0.000
Fasting glucose, mmol/L	4.94 ± 0.39	4.92 ± 0.41	0.24	0.820
Fasting insulin, pmol/L	71.08 ± 15.23	67.63 ± 14.54	2.20	0.028
HOMR-IR	2.23 ± 0.47	2.11 ± 0.42	2.56	0.011
IL6, pg/ml	67.27 ± 259.61	45.69 ± 255.26	0.79	0.426
TNFα, pg/ml	26.26 ± 85.51	16.97 ± 22.95	1.47	0.143
AG genotype	*n* = 148	*n* = 100		
Folic acid, nmol/L	31.89 ± 9.38	33.84 ± 7.62	−1.86	0.064
Vitamin B12, pg/ml	335.09 ± 107.21	340.27 ± 117.81	−0.36	0.720
Homocysteine, umol/L	12.29 ± 4.31	11.29 ± 3.07	1.99	0.047
Total protein, g/L	71.31 ± 7.81	69.38 ± 11.93	1.54	0.126
Total cholesterol, mmol/L	4.31 ± 1.06	4.24 ± 0.68	0.58	0.561
Triglyceride, mmol/L	0.93 ± 0.37	0.98 ± 0.52	−1.00	0.317
High-density lipoprotein cholesterol, mmol/L	1.65 ± 0.33	1.77 ± 0.37	−2.60	0.010
Low-density lipoprotein cholesterol, mmol/L	2.52 ± 0.73	2.45 ± 0.61	0.74	0.462
Fasting glucose, mmol/L	4.94 ± 0.37	4.94 ± 0.38	−0.05	0.960
Fasting insulin, pmol/L	71.43 ± 14.34	68.49 ± 13.09	1.84	0.067
HOMR-IR	2.24 ± 0.43	2.16 ± 0.40	1.82	0.070
IL6, pg/ml	102.91 ± 403.61	34.88 ± 120.71	2.02	0.044
TNFα, pg/ml	28.02 ± 71.11	20.87 ± 40.68	1.08	0.281
GG genotype	*n* = 30	*n* = 16		
Folic acid, nmol/L	30.15 ± 9.40	48.46 ± 80.78	−1.24	0.222
Vitamin B12, pg/ml	366.17 ± 110.06	411.75 ± 114.65	−1.32	0.194
Homocysteine, umol/L	13.84 ± 5.66	12.71 ± 3.86	0.71	0.480
Total protein, g/L	71.54 ± 10.64	72.81 ± 4.08	−0.46	0.649
Total cholesterol, mmol/L	4.56 ± 1.46	4.69 ± 1.92	−0.27	0.792
Triglyceride, mmol/L	0.87 ± 0.33	1.03 ± 0.56	−1.17	0.250
High-density lipoprotein cholesterol, mmol/L	1.67 ± 0.36	1.69 ± 0.32	−0.22	0.828
Low-density lipoprotein cholesterol, mmol/L	2.59 ± 0.87	2.48 ± 0.73	0.43	0.670
Fasting glucose, mmol/L	4.87 ± 0.33	4.94 ± 0.31	−0.92	0.360
Fasting insulin, pmol/L	70.39 ± 14.95	63.61 ± 12.91	2.19	0.032
HOMR-IR	2.18 ± 0.46	2.00 ± 0.42	1.81	0.074
IL6, pg/ml	18.62 ± 36.34	38.53 ± 180.82	−0.62	0.543
TNFα, pg/ml	37.97 ± 155.97	14.07 ± 15.96	1.08	0.283
AG/GGgenotype	*n* = 178	*n* = 116		
Folic acid, nmol/L	31.59 ± 8.56	35.86 ± 30.44	−1.76	0.079
Vitamin B12, pg/ml	340.33 ± 108.01	350.13 ± 119.48	−0.73	0.467
Homocysteine, umol/L	12.55 ± 4.58	11.49 ± 3.21	2.18	0.030
Total protein, g/L	71.34 ± 8.32	69.85 ± 11.24	1.31	0.193
Total cholesterol, mmol/L	4.36 ± 1.14	4.31 ± 0.95	0.39	0.699
Triglyceride, mmol/L	0.92 ± 0.36	0.98 ± 0.52	−1.38	0.108
High-density lipoprotein cholesterol, mmol/L	1.65 ± 0.34	1.76 ± 0.37	−2.50	0.113
Low-density lipoprotein cholesterol, mmol/L	2.52 ± 0.75	2.46 ± 0.62	0.87	0.383
Fasting glucose, mmol/L	4.93 ± 0.36	4.94 ± 0.37	−0.48	0.640
Fasting insulin, pmol/L	71.18 ± 14.46	67.56 ± 13.16	2.54	0.011
HOMR-IR	2.23 ± 0.43	2.13 ± 0.41	2.34	0.020
IL6, pg/ml	82.54 ± 353.49	35.57 ± 133.61	1.78	0.077
TNFα, pg/ml	30.42 ± 98.18	19.58 ± 37.31	1.48	0.141

*HOMA-IR* Homeostatic model assessment of insulin resistance, *IL6* Interleukin 6, *TNFα* Tumor necrosis factor α

## Discussion

We demonstrated that patients carrying the MTHFR 677CT, TT and MTRR 66AG genotypes, as well as MTHFR C677T, MTHFR A1298C and MTRR A66G alleles had a significantly higher risk of experiencing RSA. In the current study, interaction between the MTHFR C677T and A1298C polymorphism, and interaction between the MTHFR A1298C and the MTRR A66G polymorphism were associated with increased RSA risk. All the three gene SNPs except MTRR 66AG gene variant had detrimental effects on HOMA-IR. We found that compared with control group, RSA group who carried the MTHFR 677CT, TT, CT/TT genotypes and MTRR 66AG, AG/GG genotypes had detrimental effects on serum homocysteine levels, the MTHFR 677CT, CT/TT genotype carriers had favorable effects on serum folate and the MTHFR 677TT, CT/TT, 1298 AC, AC/CC genotype carriers had detrimental effects on serum LDL-C levels, the MTRR 66AG genotype carriers had lower HDL-C levels than the AA genotype carriers.

In our main effect analysis, the MTHFR C677T and MTRR A66G were the two SNPs exhibited a statistically significant association with increased recurrent spontaneous abortion risk. Besides, We also found that the MTHFR 677CT, TT, CT/TT genotypes and MTRR 66AG, AG/GG genotypes showed a higher level of homocysteine than control group and was significantly associated with recurrent spontaneous abortion. This association is biologically plausible. Homocysteine is a key factor in one-carbon folate metabolism, along with folate, is important for the proper development and growth of fetus and placenta, thus maintaining normal pregnancy [15]. It has been well documented that severe deficiency in the gene that encodes the MTHFR and MTRR enzyme reduced specific activity and increased thermolability of the enzyme, causing mild hyperhomocysteine in plasma, considered to be an important pathogenic mechanism for the development of RSA [16–18]. Besides support from biologically functional evidence, elevated plasma of homocysteine has been proven to damage the vascular endothelium and involve in placental vascular risk and endothelial dysfunction, thus lead to RSA [19].

The association between recurrent spontaneous abortion and insulin resistance is in arguement. It was reported that increased inflammatory cytokine levels such as TNFα and plasma hyperhomocysteinemia were associated with insulin resistance and endocrine abnormalities [20, 21]. Insulin resistance may have positive association with an increase of plasma hyperhomocysteinemia which may damage pregnancy by interfering with endometrial blood flow and vascular integrity leads to increase the risk of early pregnancy abortion [21].

In Mexico general populations [22] it is observed that people who carried 677 T allele may need more folate intake than those carried the C allele. Our results revealed that the MTHFR 677CT, CT/TT genotype carriers had favorable effects on serum folate, which was in accordance with the previous study demonstrated that folate deficiency related with hyperhomocysteinemia was the risk associated with recurrent abortion [23].

Besides the modest main effect of MTHFR C677T, we also observed significant effect of gene-gene interactions, which were able to amplify the modest effect of the single genetic variant, and enhance the predictive power. Individual patients with the combination of MTHFR 677 T and MTHFR 1298C had a significantly higher risk for RSA than those with the combination of MTHFR 677C and MTHFR 1298A (OR = 1.62, 95% CI: 1.28–2.04, *p* = 0.004). Logistic regression analysis showed that certain gene-gene interactions among MTHFR 1298C and MTRR 66G predict a higher risk for RSA (OR = 2.36, 95% CI: 1.228–5.297, *p* = 0.005) compared to those with the combination of MTHFR 1298A and MTRR66A. Our results were consistence with the previous studies which reported that the folate pathway gene variants and gene-gene interactions could significantly impact the occurrence of RSA [18, 24, 25].

Several studies have reported the association between the MTHFR C677T polymorphism, high homocysteine and serum lipid profiles in humans, with some indicating that the T allele was associated with unfavorable lipid profiles [26–28]. One study indicated the positive relationship between the MTHFR C677T polymorphism and the lipoprotein level in unexplained recurrent miscarriages [29]. We found that the MTHFR 677TT, CT/TT genotypes and MTHFR 1298 AC, AC/CC genotypes had detrimental effects on serum LDL-C levels and the MTRR 66GG genotype had favourable effects on serum HDL-C levels in RSA group. Our study was consistence with the study conducted by Frelut et al. who reported that MTHFR C677T gene variant was significantly increased LDL-C level [30]. Recently, Westerbuck et al. reported that sterol regulatory element binding proteins (SREBPs) can be activated by endoplasmic reticulum stress which induced by homocysteine [31]. This SREBPs was crucial for the genes responsible for cholesterol biosynthesis, uptake and intracellular accumulation. Besides support from biologically functional evidence, MTHFR-deficient mice presented hyperhomocysteinemia in mice fed control or folate-deficient diets [32]. Moreover, homocysteine was reported inversely correlated with HDL-C [9].

Publications about the influence of MTHFR A1298C mutant on serum lipid metabolic profiles were relatively rare. Chang et al. [33] found no significant associations exist between lipid profiles and MTHFR A1298C gene variants. Li et al. [9] demonstrated that MTHFR C677T and A1298C with low folate showed higher risk of low levels of high-density lipoprotein cholesterol (*p* for trend: 0.008 and 0.031). Unlike the previous studies, our data showed that MTHFR A1298C mutant was associated with higher level of LDL-C in RSA group than the healthy controls. Based on the positive association between MTHFR C677T, A1298C and serum homocysteine level [9, 24–26], and the favorable effect of homocysteine level on lipid metabolism [9], we speculate that MTHFR C677T and A1298C polymorphism and high homocysteine level interactively increased the prevalence of dyslipidemia in RSA patients.

MTRR is responsible for homocysteine remethylation. The MTRR A66G polymorphism results in its enzyme expression and affecting plasma homocysteine levels [34]. Homocysteine levels further affects serum lipid profiles [9, 34]. Many previous studies have explored the relationship between the MTRR gene polymorphisms and serum or plasma lipid profiles in humans, but with no consistence results [34–37]. For example, Misiak et al. [35] found there was no significant association between MTRR 66GG and TG or HDL-C levels in schizophrenia patients and healthy controls. But Jiang et al. [34] revealed that hypertensive patients who carried the MTRR 66GG genotype had lower serum TC and LDL-C levels than patients carried MTRR 66AA genotype. Zhi et al. [37] revealed that MTRR 66GG genotype was associated with increased risk of high TG (TG ≥1.7 mmol/L), while no significant association was found between this polymorphism and low HDL-C levels. Our data revealed that the MTRR 66AG genotype carriers had lower HDL-C levels than the AA genotype carriers, which was consistence with the previous studies reported that MTRR gene variants can affect the lipid metabolisms via plasma homocysteine levels [34–37]. But the molecular mechanism of these metabolites under conditions of folate pathway gene polymorphisms with dyslipidemia in different diseases especially RSA is not fully understood, and is worthy to be explored in the future.

### Limitation

There are some limitations to our study. First of all, the single-center design may limit the generalizability of our study results. Secondly, this case-control study is hospital-based and selection bias may exist, however, since the controls were from the same region with cases and were randomly selected from health examination population, which may reduce the effect of selection bias.

## Conclusions

In conclusion, we present the first study to date in the interactions of the MTHFR C677T, A1298C and MTRR A66G polymorphisms with the RSA risk on some serum lipid profiles. Interaction between the MTHFR C677T, A1298C and MTHFR A1298C, MTRR A66G are observed in our RSA group. Besides, all the three gene SNPs except MTRR 66AG gene variant had detrimental effects on HOMA-IR. MTHFR C677T and MTRR A66G gene variants had detrimental effects on serum homocysteine levels, while MTHFR C677T, A1298C and MTRR A66G gene variants had detrimental effects on certain serum lipid profiles. Further studies are in urgent to confirm or refute our findings in the future.

## Data Availability

We declare that the data supporting the conclusions of this article are fully described within the article, and the database is available from the first author (uters@126.com) upon reasonable request.

## Abbreviations

95%CIs: 95% confidence intervals
HDL-C: High-density lipoprotein cholesterol
HOMA-IR: Homeostatic model assessment of insulin resistance
IL6: Interleukin 6
LDL-C: Low-density lipoprotein cholesterol
MTHFR: 5–10 methylenetetrahydrofolate reductase
MTRR: Methionine synthase reductase
ORs: Odds ratios
RSA: Recurrent spontaneous abortion
SREBPs: Sterol regulatory element binding proteins
TC: Total cholesterol
TG: Triglyceride
TNFα: Tumor necrosis factor α

## References

[1] Bricker L, Farquharson RG (2002). Types of pregnancy loss in recurrent miscarriage: implications for research and clinical practice. Hum Reprod.

[2] Suga S, Tamasawa N, Kinpara I, Murakami H, Kasai N, Onuma T, Ikeda Y, Takagi A, Suda T (1998). Identification of homozygous lipoprotein lipase gene mutation in a woman with recurrent aggravation of hypertriglyceridaemia induced by pregnancy. J Intern Med.

[3] Ormazabal V, Nair S, Elfeky O, Aguayo C, Salomon C, Zuñiga FA (2018). Association between insulin resistance and the development of cardiovascular disease. Cardiovasc Diabetol.

[4] Bravo-Valenzuela NJ (2013). Elevated lipoprotein(a) in a newborn with thrombosis and a family history of dyslipidemia. Pediatr Cardiol.

[5] Wild RA (2012). Dyslipidemia in PCOS. Steroids.

[6] Luo JY, Ma YT, Yu ZX, Yang YN, Xie X, Ma X, Liu F, Li XM, Chen BD (2014). Prevalence, awareness, treatment and control of dyslipidemia among adults in northwestern China: the cardiovascular risk survey. Lipids Health Dis.

[7] Yuan X, Wang T, Gao J, Wang Y, Chen Y, Kaliannan K, Li X, Xiao J, Ma T, Zhang L, Shao Z. Associations of homocysteine status and homocysteine metabolism enzyme polymorphisms with hypertension and dyslipidemia in a Chinese hypertensive population. Clin Exp Hypertens. 2019:1-9. 10.1080/10641963.2019.1571599 30786773

[8] Li Q, Yin RX, Yan TT, Miao L, Cao XL, Hu XJ, Aung LH, Wu DF, Wu JZ, Lin WX (2011). Association of the GALNT2 gene polymorphisms and several environmental factors with serum lipid levels in the Mulao and Han populations. Lipids Health Dis.

[9] Li WX, Lv WW, Dai SX, Pan ML, Huang JF (2015). Joint associations of folate, homocysteine and MTHFR, MTR and MTRR gene polymorphisms with dyslipidemia in a Chinese hypertensive population: a cross-sectional study. Lipids Health Dis.

[10] Ellingrod VL, Miller DD, Taylor SF, Moline J, Holman T, Kerr J (2008). Metabolic syndrome and insulin resistance in schizophrenia patients receiving antipsychotics genotyped for the methylenetetrahydrofolate reductase (MTHFR) 677C/T and 1298A/C variants. Schizophr Res.

[11] Yamada H, Sata F, Saijo Y, Kishi R, Minakami H (2005). Genetic factors in fetal growth restriction and miscarriage. Semin Thromb Hemost.

[12] Wen C, Lv JF, Wang L, Zhu WF, Wan FS, Wang XZ (2015). Association of a methylene tetrahydrofolate reductase C677T polymorphism with several blood chemical levels in a Chinese population. Genet Test Mol Biomarkers.

[13] Mtiraoui N, Zammiti W, Ghazouani L, Braham NJ, Saidi S, Finan RR, Almawi WY, Mahjoub T (2006). Methylenetetrahydrofolate reductase C677T and A1298C polymorphism and changes in homocysteine concentrations in women with idiopathic recurrent pregnancy losses. Reproduction.

[14] Ikkruthi S, Rajappa M, Nandeesha H, Satheesh S, Sundar I, Ananthanarayanan PH, Harichandrakumar KT (2014). Hyperhomocysteinemia and hyperlipoproteinemia (a) in obese south Indian men: an indication for increased cardiovascular risk. Acta Physiol Hung.

[15] Bergen NE, Jaddoe VW, Timmermans S, Hofman A, Lindemans J, Russcher H, Raat H, Steegers-Theunissen RP, Steegers EA (2012). Homocysteine and folate concentrations in early pregnancy and the risk of adverse pregnancy outcomes: the generation R study. BJOG.

[16] Nelen WL, Bulten J, Steegers EA, Blom HJ, Hanselaar AG, Eskes TK (2000). Maternal homocysteine and chorionic vascularization in recurrent early pregnancy loss. Hum Reprod.

[17] Hubacek JA, Rynekrova J, Kasparova D, Adamkova V, Holmes MV, Fait T (2015). Association of MTHFR genetic variants C677T and A1298C on predisposition to spontaneous abortion in Slavonic population. Clin Chim Acta.

[18] Zhu L (2015). Polymorphisms in the methylene tetrahydrofolate reductase and methionine synthase reductase genes and their correlation with unexplained recurrent spontaneous abortion susceptibility. Genet Mol Res.

[19] van der Molen EF, Arends GE, Nelen WL, van der Put NJ, Heil SG, Eskes TK, Blom HJ (2000). A common mutation in the 5,10-methylenetetrahydrofolate reductase gene as a new risk factor for placental vasculopathy. Am J Obstet Gynecol.

[20] Akash MSH, Rehman K, Liaqat A (2018). Tumor necrosis factor-alpha: role in development of insulin resistance and pathogenesis of type 2 diabetes mellitus. J Cell Biochem.

[21] Chakraborty P, Goswami SK, Rajani S, Sharma S, Kabir SN, Chakravarty B, Jana K (2013). Recurrent pregnancy loss in polycystic ovary syndrome: role of hyperhomocysteinemia and insulin resistance. PLoS One.

[22] Friedman G, Goldschmidt N, Friedlander Y, Ben-Yehuda A, Selhub J, Babaey S, Mendel M, Kidron M, Bar-On H (1999). A common mutation A1298C in human methylenetetrahydrofolate reductase gene: association with plasma total homocysteine and folate concentrations. J Nutr.

[23] Creus M, Deulofeu R, Peñarrubia J, Carmona F, Balasch J (2013). Plasma homocysteine and vitamin B12 serum levels, red blood cell folate concentrations, C677T methylenetetrahydrofolate reductase gene mutation and risk of recurrent miscarriage: a case-control study in Spain. Clin Chem Lab Med.

[24] Zetterberg H, Zafiropoulos A, Spandidos DA, Rymo L, Blennow K (2003). Gene-gene interaction between fetal MTHFR 677C> T and transcobalamin 776C> G polymorphisms in human spontaneous abortion. Hum Reprod.

[25] Luo L, Chen Y, Wang L, Zhuo G, Qiu C, Tu Q, Mei J, Zhang W, Qian X, Wang X (2015). Polymorphisms of genes involved in the folate metabolic pathway impact the occurrence of unexplained recurrent pregnancy loss. Reprod Sci.

[26] Real JT, Martinez-Hervas S, Garcia-Garcia AB, Chaves FJ, Civera M, Ascaso JF, Carmena R (2009). Association of C677T polymorphism in MTHFR gene, high homocysteine and low HDL cholesterol plasma values in heterozygous familial hypercholesterolemia. J Atheroscler Thromb.

[27] Liu Y, Li K, Venners SA, Hsu YH, Jiang S, Weinstock J, Wang B, Tang G, Xu X (2017). Individual and joint associations of methylenetetrahydrofolate reductase C677T genotype and plasma homocysteine with dyslipidemia in a Chinese population with hypertension. Clin Appl Thromb Hemost.

[28] Zhang L, Yin RX, Liu WY, Miao L, Wu DF, Aung LH, Hu XJ, Cao XL, Wu JZ, Pan SL (2010). Association of methylenetetrahydrofolate reductase C677T polymorphism and serum lipid levels in the Guangxi Bai Ku Yao and Han populations. Lipids Health Dis.

[29] Krause M, Sonntag B, Klamroth R, Heinecke A, Scholz C, Langer C, Scharrer I, Greb RR, von Eckardstein A, Nowak-Göttl U (2005). Lipoprotein (a) and other prothrombotic risk factors in Caucasian women with unexplained recurrent miscarriage. Results of a multicentre case-control study. Thromb Haemost.

[30] Frelut ML, Emery-Fillon N, Guilland JC, Dao HH, de Courcy GP (2006). Alanine amino transferase concentrations are linked to folate intakes and methylenetetrahydrofolate reductase polymorphism in obese adolescent girls. J Pediatr Gastroenterol Nutr.

[31] Werstuck GH, Lentz SR, Dayal S, Hossain GS, Sood SK, Shi YY, Zhou J, Maeda N, Krisans SK, Malinow MR, Austin RC (2001). Homocysteine-induced endoplasmic reticulum stress causes dysregulation of the cholesterol and triglyceride biosynthetic pathways. J Clin Invest.

[32] Mikael LG, Wang XL, Wu Q, Jiang H, Maclean KN, Rozen R (2009). Hyperhomocysteinemia is associated with hypertriglyceridemia in mice with methylenetetrahydrofolate reductase deficiency. Mol Genet Metab.

[33] Chang YH, Fu WM, Wu YH, Yeh CJ, Huang CN, Shiau MY (2011). Prevalence of methylenetetrahydrofolate reductase C677T and A1298C polymorphisms in Taiwanese patients with type 2 diabetic mellitus. Clin Biochem.

[34] Jiang S, Zhao R, Pan M, Venners SA, Zhong G, Hsu YH (2014). Associations of MTHFR and MTRR polymorphisms with serum lipid levels in Chinese hypertensive patients. Clin Appl Thromb Hemost.

[35] Misiak B, Łaczmański Ł, Słoka NK, Szmida E, Piotrowski P, Loska O, Ślęzak R, Kiejna A, Frydecka D (2016). Metabolic dysregulation in first-episode schizophrenia patients with respect to genetic variation in one-carbon metabolism. Psychiatry Res.

[36] Yang B, Fan S, Zhi X, Wang D, Li Y, Wang Y, Wang Y, Wei J, Zheng Q, Sun G (2014). Associations of MTHFR C677T and MTRR A66G gene polymorphisms with metabolic syndrome: a case-control study in northern China. Int J Mol Sci.

[37] Zhi X, Yang B, Fan S, Wang Y, Wei J, Zheng Q, Sun G (2016). Gender-specific interactions of MTHFR C677T and MTRR A66G polymorphisms with overweight/obesity on serum lipid levels in a Chinese Han population. Lipids Health Dis.

